# Quantifying statistical cure in unresectable locally advanced esophageal squamous cell carcinoma treated with radiotherapy-based regimens: a cure model analysis with SEER validation

**DOI:** 10.3389/fonc.2026.1768800

**Published:** 2026-03-26

**Authors:** Nan Wang, Xiangyang Zhang, Xiaoli Zheng, Xiaohui Wang, Ying Jiang, Zhuangzhuang Zhang, Yibo Zhao, Yumeng Xiao, Shuhan Yang, Longxin Zhu, Xiance Tang, Baosheng Li, Yexiong Li, Nan Bi, Hong Ge

**Affiliations:** 1 Department of Radiology, The First Affiliated Hospital of Zhengzhou University, Zhengzhou, Henan, China; 2 Department of Radiation Oncology, The Affiliated Cancer Hospital of Zhengzhou University & Henan Cancer Hospital, Zhengzhou, Henan, China; 3 Department of Oncology, Xiangya Hospital of Central South University, Changsha, Hunan, China; 4 Department of Radiation Oncology, National Cancer Center/National Clinical Research Center for Cancer/Cancer Hospital, Chinese Academy of Medical Sciences and Peking Union Medical College, Beijing, China; 5 Department of Radiation Oncology, Shandong Cancer Hospital and Institute, Shandong First Medical University and Shandong Academy of Medical Sciences, Jinan, China

**Keywords:** chemoradiotherapy, cure fraction, cure model, cure point, immune checkpoint inhibitors, radiotherapy, seer database, statistical cure

## Abstract

**Purpose:**

This study aimed to quantify the statistical cure in patients with unresectable locally advanced esophageal squamous cell carcinoma (LA-ESCC) treated with definitive radiotherapy (RT)-based strategies and to explore the independent prognostic factors of cure.

**Methods and Materials:**

This retrospective study analyzed 801 patients with unresectable LA-ESCC at Affiliated Cancer Hospital of Zhengzhou University from 2014 to 2023. All patients received definitive RT and were stratified into chemoradiotherapy (CRT, n=689) or CRT combined with immunity checkpoint inhibitors (CRT+ICIs, n=112) based on treatment received. A relative survival (RS)-based mixture cure modeling methodology was used to estimate the cure fraction and cure point. The model was externally validated using a dataset of 5,000 matched patients from the SEER database.

**Results:**

Our model estimated a cure fraction of 10.4% and a cure point of 6.7 years. Subset analysis indicated that patients treated with CRT+ICIs had a higher cure fraction than those treated with CRT (30.6% vs 10.9%), with a shorter time to cure (3.9 vs 7.1 years). Validation with the SEER dataset showed a comparable cure fraction (10.8%) but a longer cure point (9.3 years). Multivariable analysis suggested that the favorable independent prognostic factors of statistical cure included a higher BMI (*β*, 0.10; 95% CI, 0.01 to 0.19; p=0.039) and the CRT+ICIs regimen (*β*, 2.18; 95% CI, 0.91 to 3.44; p<0.001). Factors associated with a lower probability of cure were age ≥65 years (*β* -0.04; 95% CI, -0.09 to 0.00; p=0.032) and chronic comorbidities (*β*, -1.10; 95% CI, -2.14 to -0.05; p=0.040).

**Conclusions:**

Statistical cure is achievable in unresectable LA-ESCC patients receiving RT-based regimens. However, the traditional 5-year overall survival (OS) insufficiently reflects long-term survival, indicating that follow-up in the CRT group should be at least 7 years. Incorporation of ICIs facilitated curative potential and shortened cure point, supporting a 4-year OS surrogate endpoint for CRT+ICIs. These findings emphasize the value of integrating cure models into clinical practice to optimize individualized treatment and surveillance strategies.

## Introduction

Esophageal squamous cell carcinoma (ESCC) is a globally prevalent malignancy associated with unfavorable long-term outcomes, particularly for patients with unresectable locally advanced ESCC (LA-ESCC) (1, 2). Definitive chemoradiotherapy (CRT) has long been the cornerstone of treatment for ESCC, with a 5-year overall survival (OS) rate of about 30% (3). However, there is little research on whether any patient subsets can be considered truly “cured”. Moreover, specific factors that could influence curability of ESCC patients have not been well characterized.

A major challenge in unresectable LA-ESCC research is the lack of standardization for estimating long-term survival (4). Although 5-year OS is widely used as a surrogate endpoint for cure, 10-year follow-up data from landmark studies reveal a continued decline in survival rate beyond this timeframe, casting doubt on its reliability (5, 6). This discrepancy raises two key questions: whether the 5-year OS accurately reflects “cure” in LA-ESCC, and when post-diagnosis survival rates converge with those of the general population. Therefore, the usage of mature analytical methods is necessary to accurately estimate the statistical cure.

Statistical cure is achieved when the mortality rate of a patient cohort becomes similar to that of the general population, after adjusting for demographic factors such as age, sex, and calendar year (7). The cure model can estimate statistical cure by determining when the relative survival (RS) curve reaches a stable plateau, signifying that disease-specific excess mortality has ceased (8, 9). This analysis yields two key elements: the cure fraction, defined as the proportion of patients who achieve this curative state, and the cure point, defined as the time at which the plateau begins. Of the two elements, cure point can serve as a robust surrogate endpoint for long-term survival (8). Although statistical cure models have been validated in various malignancies, including nasopharyngeal carcinoma (NPC), non-small-cell lung cancer (NSCLC), and extranodal nasal-type NK/T-cell lymphoma (ENKTCL), its applicability and clinical value in unresectable LA-ESCC, particularly in the era of immunotherapy, have not been well-explored (9–14).

To bridge this gap, we aimed to not only quantify the cure fraction and cure point in patients with unresectable LA-ESCC, but to also identify factors associated with statistical cure. The robustness of our primary findings was subsequently validated using an independent cohort from the Surveillance, Epidemiology, and End Results (SEER) database, which was selected based on identical inclusion criteria.

## Materials and methods

### Study design, data collection and eligibility criteria

This retrospective single-center cohort study enrolled patients with unresectable LA-ESCC that received definitive radiotherapy (RT)-based regimens at Affiliated Cancer Hospital of Zhengzhou University from 2014 to 2023. A total of 801 patients were included based on the following criteria: (1) histologically confirmed ESCC; (2) deemed inoperable after comprehensive clinical evaluation; (3) received definitive RT with a total dose ≥ 50.4 Gy, combined with chemotherapy with or without immunotherapy; (4) no observable distant metastases at diagnosis (M0); and (5) no prior antitumor therapy. All tumors were staged as Stage II, III, or IVA according to the 8th edition of the American Joint Committee on Cancer (AJCC) staging system. Baseline data, including age at diagnosis, sex, Karnofsky performance status (KPS), body mass index (BMI), major comorbidities, tumor location, TNM stage, and treatment details, were extracted from electronic medical records. This study was approved by the Ethics Committee of Affiliated Cancer Hospital of Zhengzhou University.

### Treatment modalities

The patients were stratified into two treatment groups: definitive CRT (n=689, 86.0%) and CRT plus immunity checkpoint inhibitors (ICIs) (n=112, 14.0%). The foundational CRT regimen for all patients consisted of conventionally fractionated radiotherapy (1.8-2.0 Gy per fraction, 5 fractions per week; total dose 50.4–66 Gy) delivered with 2–4 cycles of a platinum-based dual-drug chemotherapy. For the CRT+ICIs group, immunotherapy consisted of an anti-PD-1 or anti-PD-L1 antibody administered intravenously every 3 weeks, continued for up to two years or until the detection of disease progression or unacceptable toxicity.

### Statistical analysis

#### Relative survival and cure modeling

The primary endpoint of RS was used to estimate disease-specific survival without dependence on cause-of-death. In addition, OS was defined as the time from diagnosis until death from any cause. RS at time *t* was calculated as:


$$RSt=\frac{S_{observed}(t)}{S_{expected}(t)}$$

where $S_{observed}(t)$ represents the observed OS, estimated using the Kaplan-Meier method, and $S_{expected}(t)$ is the expected survival derived from general population life tables matched for age, sex, calendar year. Of note, national life tables for China were used for the institutional cohort, and U.S. life tables were used for the SEER validation cohort. To estimate the cure fraction and cure point, mixture cure models were fitted using 6 parametric distributions: Exponential, Weibull, Log-normal, Weibull-Exponential mixture, Weibull–Weibull mixture, and Generalized Modified Weibull. The Weibull–Weibull mixture model was selected for primary analysis based on the lowest Akaike Information Criterion (AIC) value (-2461.8; **Supplementary Figure 1f**). To assess model fit, the observed Kaplan-Meier survival curves were plotted against the fitted RS curves for visual comparison. Additionally, standardized mortality ratios (SMRs) were calculated as the ratio of observed to expected deaths for specific time intervals and plotted along with their pointwise 95% CIs for quantitative evaluation. Operationally, the cure point was defined as the time when the patient cohort’s SMR was no longer statistically different from 1.0. The SMR-based result was then used to corroborate the plateau identified on the RS curves, thereby providing robust evidence for the achievement of statistical cure.

#### Model performance and external validation

Time-dependent receiver operating characteristic (ROC) analysis was used to evaluate the model’s discriminatory performance at various follow-up time points. The time-dependent area under the curve (AUC) was calculated to account for censored data, with values >0.7, >0.8, and >0.9 considered to indicate acceptable, good, and excellent discrimination, respectively. An independent validation cohort of 5,000 patients with ESCC that received definitive CRT without surgery and with no observable distant metastases was selected from the SEER database (2000–2022). We applied the same Weibull-Weibull mixture cure model to the SEER cohort to estimate the cure fraction and cure point. The results were compared to those obtained from our institutional cohort to evaluate generalizability.

All analyses were performed using R (v4.1.2) and STATA (v16). Two-sided p-values <0.05 were considered statistically significant.

## Results

### Patient characteristics and follow up

The institutional cohort included 801 patients with a median age of 67 years (interquartile range [IQR], 60–72 years); 69.4% of patients were male. Approximately 45.3% had ≥1 chronic comorbidity (e.g., diabetes, hypertension, and coronary artery disease). According to the standard of AJCC 8th edition staging, 265 (33.1%) were stage II, 420 (52.4%) were stage III, and 116 (14.5%) were stage IVA. In comparison, the SEER validation cohort included 5,000 patients with a higher proportion of males (83.9%, n=4,196). The Stage distribution was 1,988 (39.8%) stage II, 2,834 (56.7%) stage III, and 178 (3.5%) stage IV (**Supplementary Table 1**). A comprehensive summary of baseline characteristics for both cohorts is provided in **Table 1** and **Supplementary Table 1**, respectively.

**Table 1:** Baseline characteristics.

Characteristics	Overall	Treatment modality		P-value
		CRT	CRT with ICIs	
Number of cases	801 (100%)	689 (86.0%)	112 (14.0%)	
Age[IQR]	67.0 [60.0, 72.0]	67.0 [60.0, 72.0]	68.00 [60.0, 72.3]	<0.001
< 65	307 (38.2%)	265 (38.5%)	42 (37.5%)	
≥ 65	494 (61.8%)	424 (61.5%)	70 (62.5%)	
Sex				0.995
Male	556 (69.4%)	478 (69.4%)	78 (69.6%)	
Female	245 (30.6%)	211 (30.6%)	34 (30.4%)	
KPS				0.568
≤ <70	50(6.2%)	41 (6.0%)	9 (8.0%)	
>70	751 (93.8%)	648 (94.0%)	103 (92.0%)	
Chronic conditions				0.044
No	438 (54.7%)	415 (60.2%)	57 (50.9%)	
Yes	363 (45.3%)	274 (39.8%)	55 (49.1%)	
BMI[IQR]	22.7 [20.5, 24.7]	22.7[20.7,24.7]	22.5 [20.0, 25.3]	0.286
Smoke				0.158
No	438 (54.7%)	382 (55.4%)	56 (50.0%)	
Yes	363 (45.3%)	307 (44.6%)	56 (50.0%)	
Alcohol				0.06
No	494 (61.7%)	423 (61.4%)	71 (63.4%)	
Yes	307 (38.3%)	266 (38.6%)	41 (36.6%)	
Location				0.132
Cervical	87(10.9%)	81 (11.8%)	6 (5.3%)	
Upper	235 (29.3%)	204 (29.6%)	31 (27.7%)	
Middle	295 (36.8%)	248 (36.0%)	47 (42.0%)	
Lower	184 (23.0%)	156 (22.6%)	28 (25.0%)	
T				0.233
1	2 (0.3%)	2 (0.3%)	0 (0.0%)	
2	171 (21.3%)	149 (21.6%)	22 (19.6%)	
3	534 (66.7%)	455 (66.0%)	79 (70.6%)	
4	94 (11.7%)	83 (12.1%)	11 (9.8%)	
N				0.075
0	201 (25.1%)	173 (25.1%)	28 (25.0%)	
1	399 (49.8%)	349 (50.7%)	50 (44.6%)	
2	175 (21.9%)	145 (21.0%)	30 (26.8%)	
3	26 (3.2%)	22 (3.2%)	4 (3.6%)	
8th AJCC Stage				0.024
II	265 (33.1%)	229 (33.2%)	36 (32.1%)	
III	420 (52.4%)	358 (52.0%)	62 (55.4%)	
IVA	116 (14.5%)	102 (14.8%)	14 (12.5%)	
Outcome				<0.001
alive	283 (35.3%)	224 (32.5%)	59 (52.7%)	
dead	518 (64.7%)	465 (67.5%)	53 (47.3%)	

ICIs, immune checkpoint inhibitor; IQR, interquartile range; KPS, Karnofsky Performance Status; BMI, Body Mass Index; AJCC, American Joint Committee on Cancer; RT, radiotherapy; CRT, chemo-radiotherapy.

### Statistical cure in unresectable LA-ESCC patients

**Figure 1:**
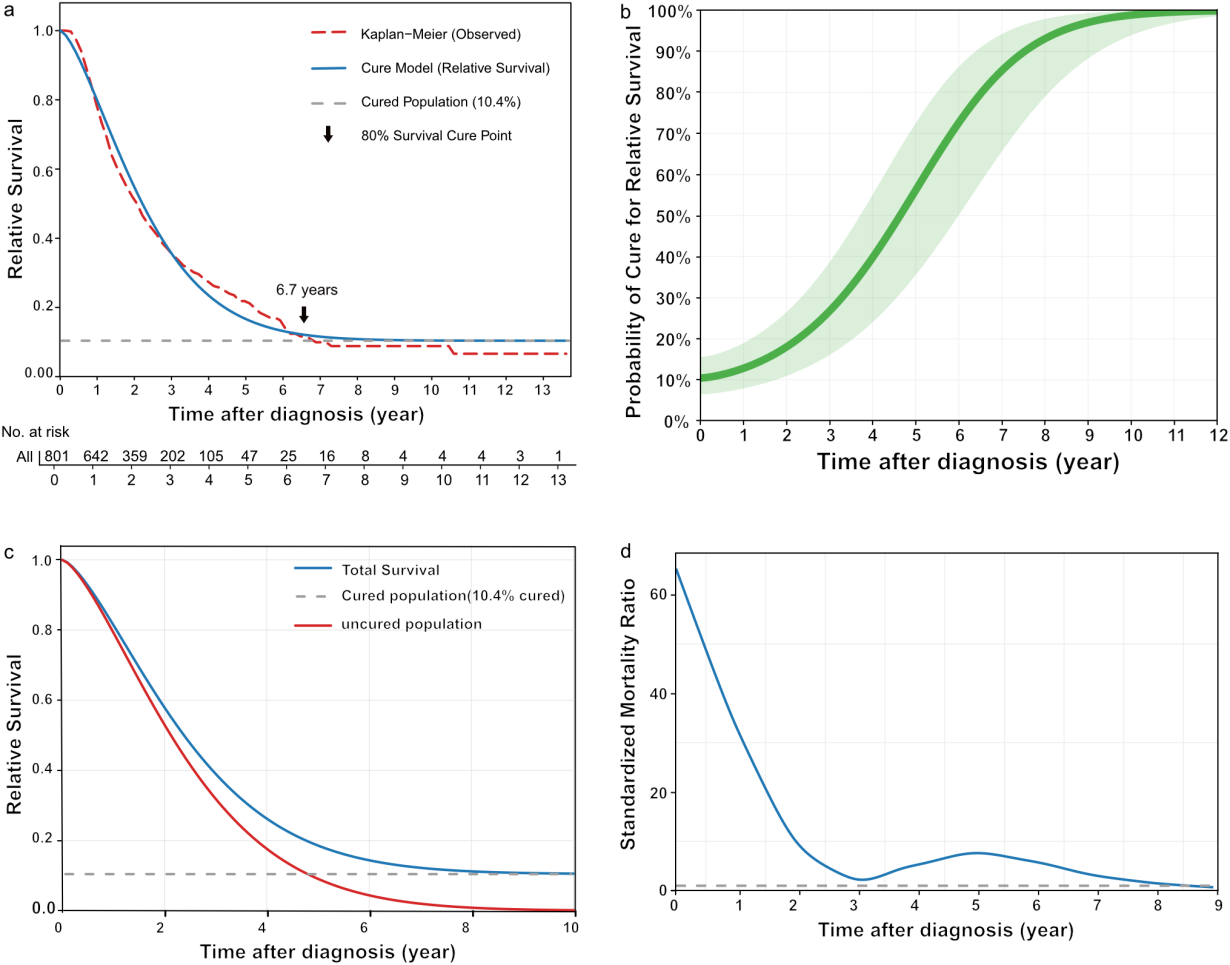
Cure model analysis for the unresectable esophageal squamous cell carcinoma patients. **(a)** Relative survival (RS) curve fitted by a mixture cure model. The red curve represents the Kaplan-Meier observed survival data, the blue curve represents the fitted cure model, and the gray dashed line indicates the estimated cure fraction. The arrow indicates the cure point at which 80% of surviving patients are considered cured. **(b)** Time-dependent cure probability. The solid line represents the mean value and the shaded area indicates the 95% confidence interval (CI). **(c)** RS curves for the total population (blue), uncured population (red), and cured population (gray dashed line). **(d)** The trend of the standardized mortality ratio (SMR) over time. The horizontal dashed line indicates SMR = 1.0.

The mixture cure model was successfully fitted to the institutional cohort, identifying a statistically “cured” patient subset. The RS curve reached a stable plateau at approximately 6.7 years, which defined the cure point and corresponded to a cure fraction of 10.4% (**Figure 1A**). This finding indicates that beyond this time point, the excess mortality risk became negligible, and the long-term survival of this cured cohort began to become similar that of the general population. Although the probability of cure increased with continued follow-up, fewer than 60% of patients surviving to 5 years met the definition of statistical cure (**Figure 1B**). This result strongly suggests that 5-year OS is an inadequate surrogate for cure, as a substantial proportion of patients remain at risk for late recurrence or disease-specific mortality beyond this conventional landmark. Analysis of the RS curves revealed a steep and continuous decline for the uncured subpopulation, in contrast to a stabilizing plateau that emerged for the overall cohort after approximately 7 years, visually confirming the existence of a cured fraction (**Figure 1C**). The SMR demonstrated a dynamic, multiphasic pattern over time (**Figure 1D**). Initially, the SMR was extremely high (>60), reflecting a markedly elevated risk of early mortality. A pronounced decline was subsequently observed over the first 3 years, with the SMR approaching 1.0 (a temporary normalization of risk). However, a second peak of excess mortality was detected around the 5-year mark (SMR≈7.0) before the ratio finally returned to parity with the general population by year 8. This pattern of excess mortality, particularly the resurgence after 5 years, calls into question the validity of 5-year OS as a sufficient proxy for cure. The time-dependent AUC values between 2 and 8 years ranged from 0.80 to 0.88, with narrow 95% CIs, indicating robust and reliable predictive performance of the model (**Supplementary Figure 2**).

### Curability in the SEER validation cohort

**Figure 2:**
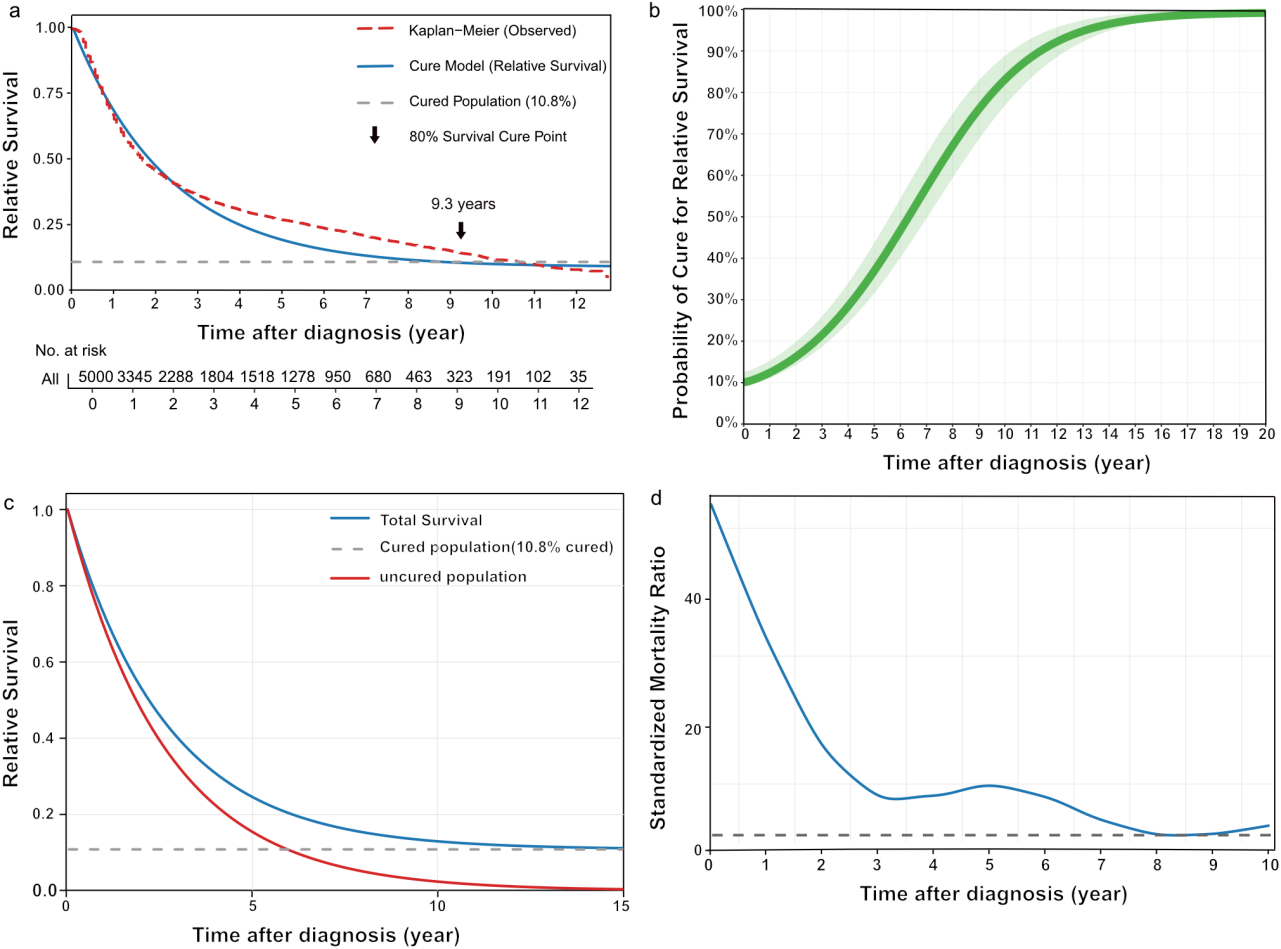
Cure model analysis for the SEER cohort. **(a)** RS curve fitted by a mixture cure model. The red curve represents the Kaplan-Meier observed survival data, the blue curve represents the fitted cure model, and the gray dashed line indicates the estimated cure fraction. The arrow indicates the cure point at which 80% of surviving patients are considered cured. **(b)** Time-varying probability of cure, with the solid line representing the mean estimate and the shaded area indicating the 95% confidence interval. **(c)** RS for the total population (blue), uncured population (red), and cured population (gray dashed line). **(d)** The trend of the SMR over time. The horizontal dashed line indicates SMR = 1.0.

Application of the identical model to the SEER cohort yielded a cure fraction of 10.8%, which was consistent with the 10.4% observed in the institutional cohort; however, the estimated cure point was substantially longer, at approximately 9.3 years (**Figure 2a**). The time-dependent cure probability surpassed 80% by 9 years, whereas only approximately 40% of survivors were deemed cured at 5 years (**Figures 2B, C**). The temporal trends in SMR were analogous to those of the institutional cohort, with a secondary peak at 5 years, where the SMR was approximately fivefold higher than that of the general population (**Figure 2D**). Taken together, these consistent findings highlight the clinical necessity of extended follow-up to accurately capture long-term outcomes.

### Cure fraction by treatment modality and patient characteristics

**Figure 3:**
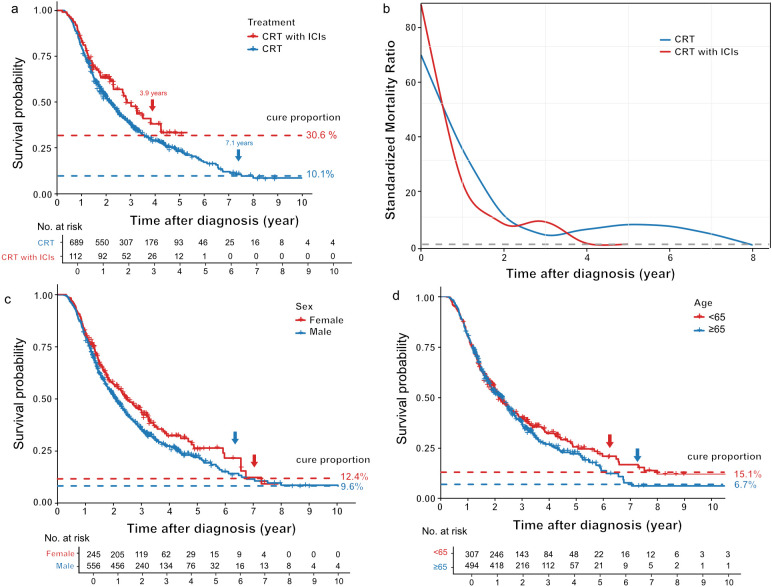
Cure model analysis across clinical subgroups. **(a)** RS curves stratified by treatment modality, including CRT (blue line) and CRT+ICI (red line). Horizontal dashed lines indicate estimated cure fractions. The arrows highlight the cure points. **(b)** Time-dependent SMR by treatment group. CRT is represented by the blue line and CRT plus ICIs by the red line. **(c)** Survival curves stratified by sex, with females in red and males in blue. **(d)** Age-stratified survival curves in the institutional. Patients aged <65 years are represented by red lines, and those aged ≥65 years by blue lines.

In the institutional cohort, subgroup analysis revealed a significant therapeutic advantage for patients that received ICIs (**Figure 3A**). The estimated cure fraction was nearly threefold higher in the CRT+ICIs group compared to the CRT group (30.6% vs. 10.9%, respectively). Furthermore, the time to achieve a statistical cure was substantially shorter, with a cure point of 3.9 years compared to 7.1 years for those treated with CRT. These results indicate that the addition of ICIs improves curative outcomes. The SMR trajectories for the two treatment groups followed a similar trend, but differed substantially in their timelines (**Figure 3B**). For the CRT group, an initial high SMR declined sharply over the first 3 years, followed by a secondary peak at approximately 5 years (SMR≈7.0), and normalization (SMR≈1.0) around year 8. In contrast, the SMR for the CRT+ICIs group followed an accelerated timeline: the secondary peak occurred earlier at 3 years (SMR≈8.0), and normalization was achieved by year 4. This accelerated normalization aligns with the 3.9-year cure point for the CRT+ICIs group, supporting 4-year OS as a potential surrogate endpoint for cure. Conversely, the prolonged SMR elevation in the CRT group supplements the validity of 5-year OS and suggests that a follow-up of at least 7 years is necessary for an accurate assessment of long-term outcomes. These results underscore the capacity of immunotherapy to mitigate long-term excess cancer-related mortality.

Subgroup analyses identified several patient characteristics associated with the probability of cure across both cohorts. Female patients exhibited a higher cure fraction than males (12.5% vs. 9.9%; **Figure 3C**). Similarly, age was a significant factor, with patients aged <65 years having a more than twofold higher cure fraction compared to those ≥65 years (15.1% vs. 6.7%; **Figure 3D**). The cure fraction also correlated with clinical stage, decreasing from 14.2% for stage II to 10.9% for stage III. For patients with stage IVA disease, however, no definitive cure fraction could be estimated, indicating a dismal prognosis (**Supplementary Figure 3D**). These trends were consistently observed in the SEER validation cohort (**Supplementary Figures 3A–C**).

### Multivariable regression analysis

**Figure 4:**
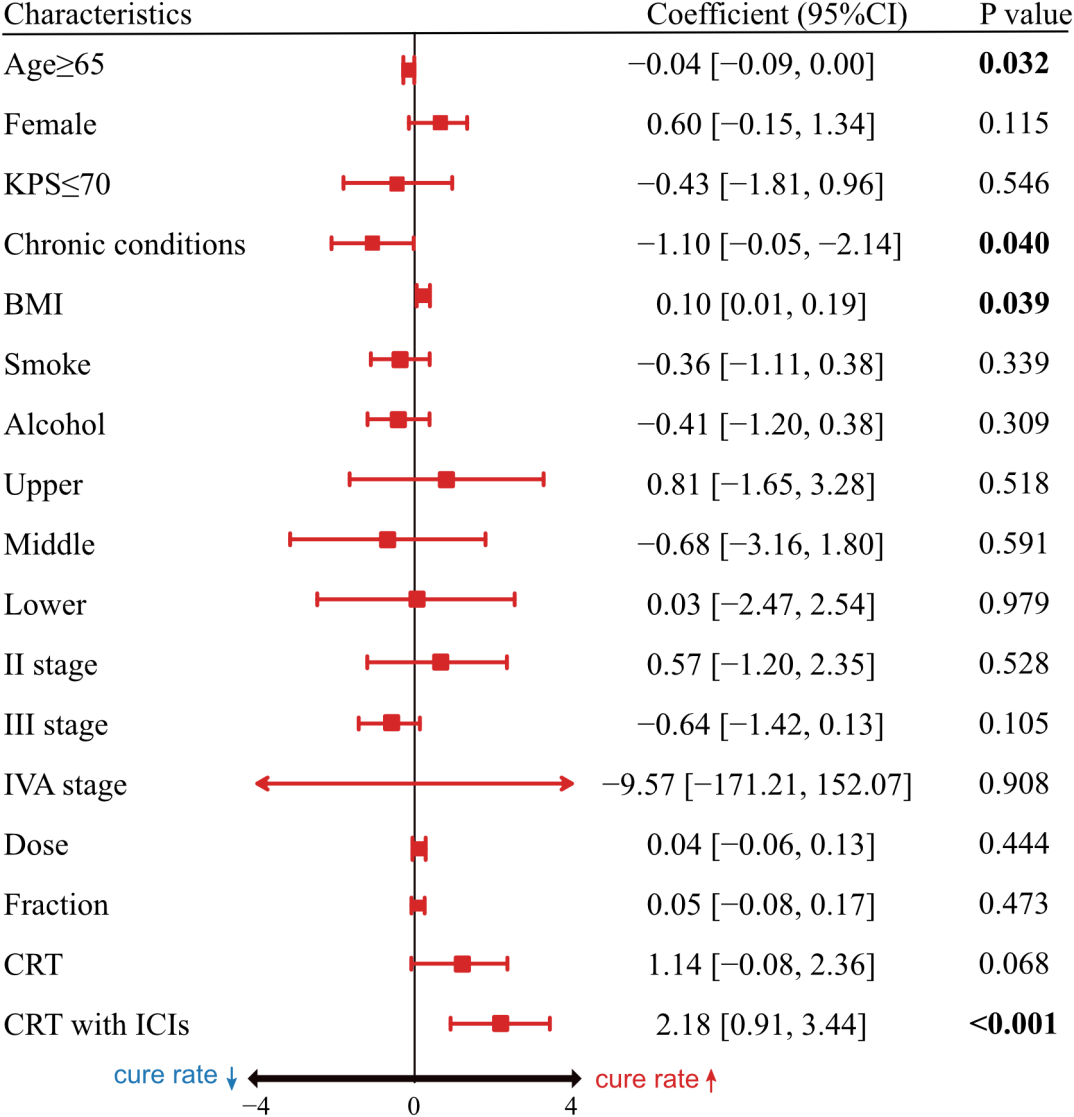
Multivariable cure model analysis of statistical cure. Forest plot displays estimated coefficients and 95% CI for each clinical variable. Markers to the right of the vertical line indicate higher cure rate; markers to the left indicate lower cure rate. PS, Karnofsky Performance Status; BMI, body mass index; CRT, chemoradiotherapy; ICIs, immune checkpoint inhibitor.

To identify clinical factors associated with statistical cure, we conducted a multivariable regression analysis, with the results summarized in a forest plot (**Figure 4**). The model included the following characteristics: age, sex, KPS, comorbidities, BMI, smoking and alcohol history, tumor location, AJCC 8th edition stage, radiation dose and fractionation, and treatment modality. The results showed that two factors remained significantly associated with a lower probability of cure: the presence of chronic comorbidities (β, -1.10;95% CI, -2.14 to -0.05; p= 0.040) and age≥65 years (β, -0.04; 95% CI, -0.09 to 0.00; p= 0.032). Conversely, a higher BMI (β, 0.10; 95% CI, 0.01 to 0.19; p= 0.039) and treatment with CRT plus ICIs (β, 2.18; 95% CI, 0.91 to 3.44; p<0.001) were independently associated with a higher probability of cure.

## Discussion

In this study, we demonstrate that statistical cure is achievable in patients with unresectable LA-ESCC undergoing radiotherapy-based treatment, though the cure point extends beyond the 5-year landmark. The robustness of this finding was confirmed through external validation using a cohort derived from the SEER database. Our analysis identified treatment with CRT plus ICIs as the strongest factor for favorable prognosis, as it both increased the cure fraction and shortened the cure point. However, advanced age and the presence of comorbidities were independent adverse prognostic factors. These insights elucidate the characteristics of long-term survival in this population and provide a data-driven basis for optimizing clinical management.

Our primary finding–that the statistical cure point for unresectable LA-ESCC is achieved at 6.7 and 9.3 years in our institutional and SEER cohorts, respectively–directly challenges the credibility of 5-year OS as a surrogate for cure. This extended time to cure is consistent with findings in other challenging malignancies, such as metastatic colorectal cancer (9). In contrast, cancers with more favorable prognoses, including ENKTCL and NPC, exhibit much shorter cure points (12, 13). This demonstrates that the adequacy of 5-year OS as a surrogate for cure is highly dependent on the specific malignancy and its inherent biological aggressiveness, rendering it an insufficient marker for poor-prognosis diseases such as unresectable LA-ESCC (15).

Although the advent of immunotherapy has shown potential for improving survival in LA-ESCC, its ability to increase the rate of statistical cure has remained unclear (16–22). In this context, our study provides key evidence, demonstrating that the addition of ICIs confers a significant therapeutic benefit, not only enhancing the cure fraction but also reducing the time to cure (23). This finding is consistent with recent LA-ESCC clinical trials; for example, studies by Lian et al., Ma et al., and Park et al. have all reported improved survival rates and reduced mortality risks with the inclusion of immunotherapy (24–26). Furthermore, our results echo those documented in unresectable stage III NSCLC, where a similar increase in the cure fraction has been observed with the addition of ICIs (14).

Our findings challenge the universal utility of the 5-year OS landmark as a “one-size-fits-all” surrogate for cure in LA-ESCC. Instead, we propose a treatment-specific paradigm: 4-year OS serves as a novel surrogate endpoint for patients receiving CRT + ICIs, potentially alleviating patient anxiety and optimizing resource utilization. In contrast, the persistent risk of late mortality in CRT patients necessitates extending surveillance to at least 7 years, which emphasizes the need for counseling on recurrence risk beyond the conventional 5-year threshold. Multivariable analysis identified key prognostic factors, with higher BMI independently predicting increased cure probability, and chronic comorbidities being associated with a lower probability. These findings enhance prognostic precision and highlight the value of comprehensive care—including nutritional optimization and proactive comorbidity management—in improving long-term outcomes.

This analysis represents the first large-scale application of statistical cure modeling in unresectable LA-ESCC, bolstered by external validation to enhance its generalizability. However, several limitations should be acknowledged, including the lack of predictive biomarkers for ICIs response, the relatively short median follow-up in the CRT plus ICIs cohort, and the inherent constraints of a retrospective study design. Future prospective trials should prioritize the identification of predictive biomarkers, the optimization of CRT-immunotherapy combinations, and the inclusion of diverse ethnic populations.

In conclusion, this study quantifies statistical cure in patients with unresectable LA-ESCC and guides tailored surveillance strategies for different treatment regimens. Integrating statistical cure models into clinical practice provides valuable insight into long-term survival, facilitating an optimized, patient-centered therapeutic, and surveillance framework.

## Data Availability

The original contributions presented in the study are included in the article/**Supplementary Material**. Further inquiries can be directed to the corresponding authors.

## Supplementary material

Supplementary Figure 1 Model fitting and performance evaluation of RS curves.**(a–e)** Observed RS curves (red lines) compared with fitted curves (blue lines) from different cure models: Exponential model **(a)**, Weibull–Exponential mixture model **(b)**, Weibull model **(c)**, Log-normal model **(d)**, and Generalized modified Weibull model **(e)**. **(f)** The AIC values of 6 parametric distributions.

Supplementary Figure 2 Time-dependent area under the curve (AUC) with 95% CI. The dashed red line indicates an AUC of 0.5.

Supplementary Figure 3 Cure model analysis across clinical subgroups in SEER cohort. **(a)** Survival curves stratified by sex, with females in red and males in blue. **(b)** Age-stratified survival curves. Patients aged <65 years are represented by red lines, and those aged ≥65 years by blue lines. **(c, d)** Survival curves by AJCC stage in the SEER **(c)** and institutional **(d)** cohorts. Stage II, III, and IVA patients are denoted by red, yellow, and blue lines, respectively.

Supplementary Table 1 Baseline characteristics of the SEER subset (2000–2022).
