# Supplementary figures and images for: Quantifying statistical cure in unresectable locally advanced esophageal squamous cell carcinoma treated with radiotherapy-based regimens: a cure model analysis with SEER validation
Source: Front Oncol. 2026 Mar 26;16:1768800. doi: 10.3389/fonc.2026.1768800 (PMC13061866; doi:10.3389/fonc.2026.1768800)

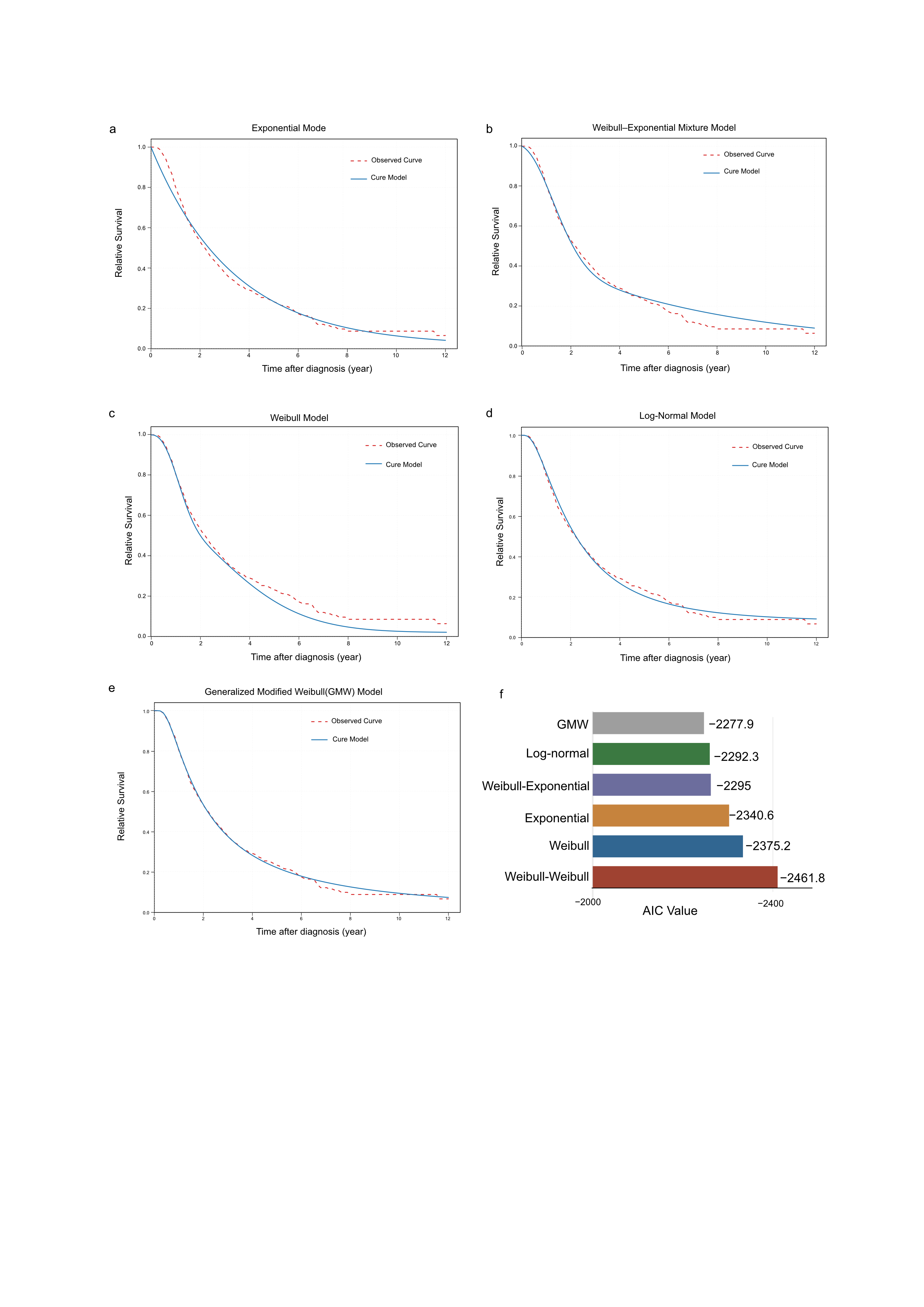

Supplement: Supplementary Figure 1 — Model fitting and performance evaluation of RS curves.(a–e) Observed RS curves (red lines) compared with fitted curves (blue lines) from different cure models: Exponential model (a), Weibull–Exponential mixture model (b), Weibull model (c), Log-normal model (d), and Generalized modified Weibull model (e). (f) The AIC values of 6 parametric distributions. [file Image1.tiff]

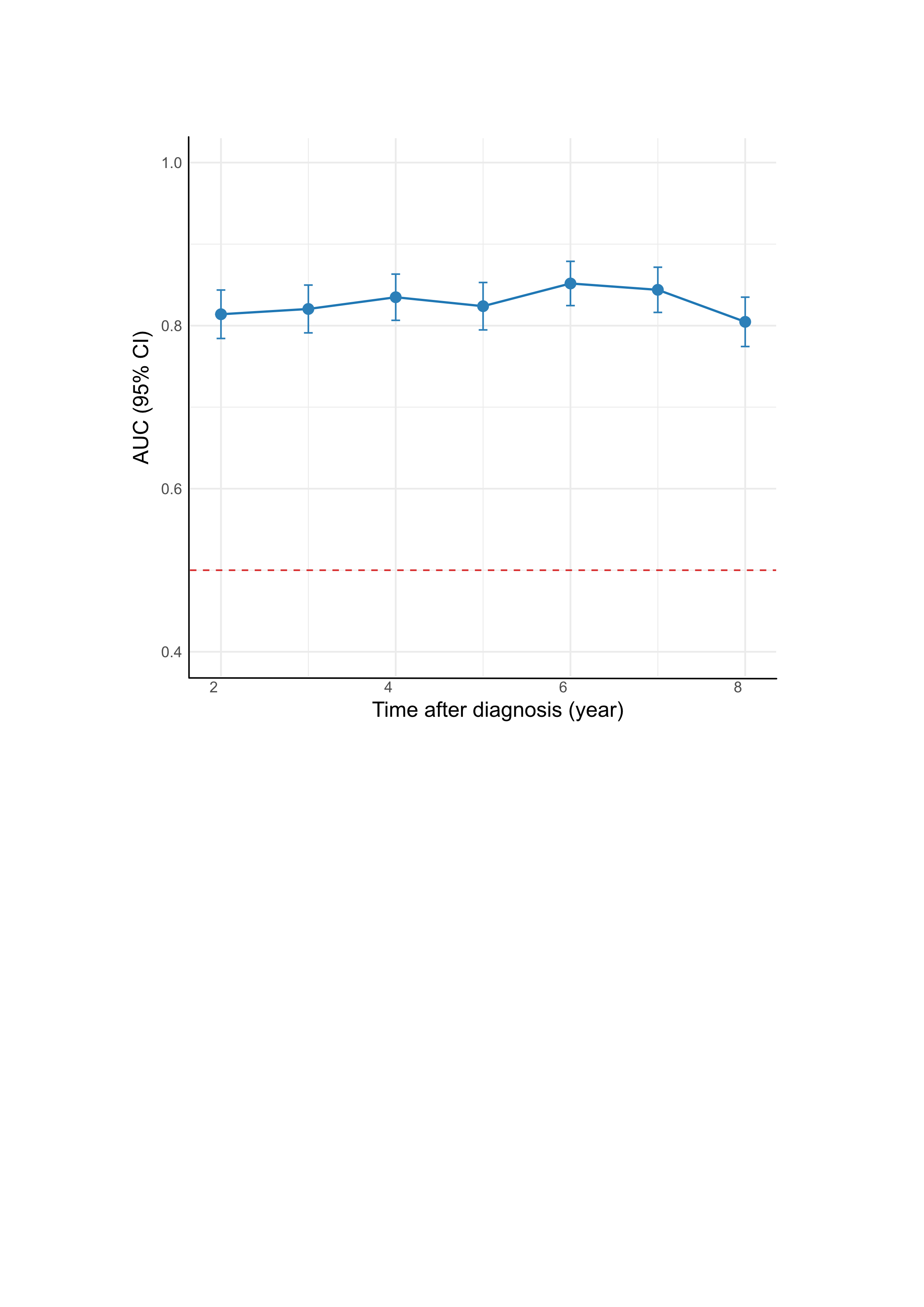

Supplement: Supplementary Figure 2 — Time-dependent area under the curve (AUC) with 95% CI. The dashed red line indicates an AUC of 0.5. [file Image2.tiff]

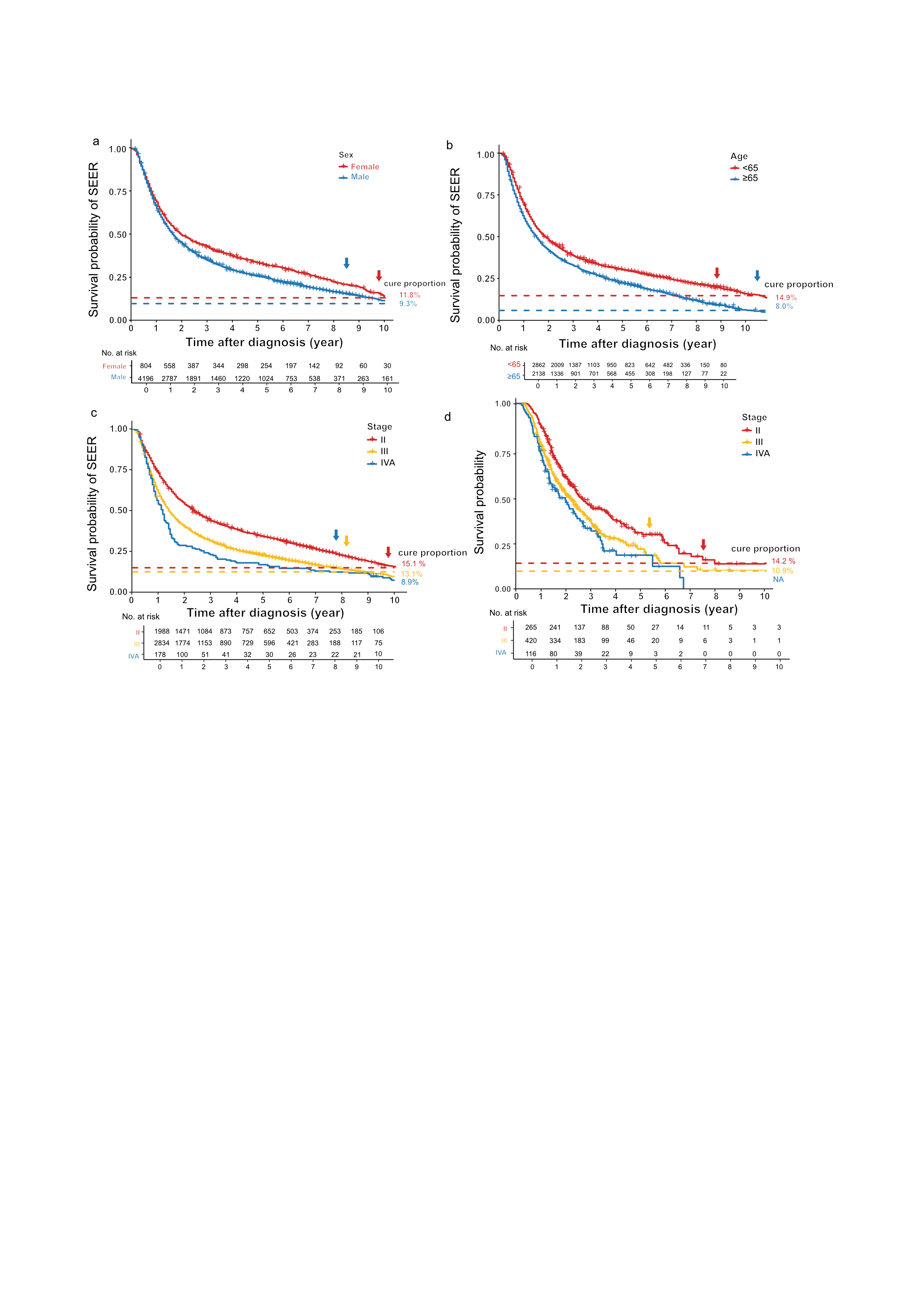

Supplement: Supplementary Figure 3 — Cure model analysis across clinical subgroups in SEER cohort. (a) Survival curves stratified by sex, with females in red and males in blue. (b) Age-stratified survival curves. Patients aged <65 years are represented by red lines, and those aged ≥65 years by blue lines. (c, d) Survival curves by AJCC stage in the SEER (c) and institutional (d) cohorts. Stage II, III, and IVA patients are denoted by red, yellow, and blue lines, respectively. [file Image3.tiff]
