# Supplementary material for: Quantifying statistical cure in unresectable locally advanced esophageal squamous cell carcinoma treated with radiotherapy-based regimens: a cure model analysis with SEER validation
Source: Front Oncol. 2026 Mar 26;16:1768800. doi: 10.3389/fonc.2026.1768800 (PMC13061866; doi:10.3389/fonc.2026.1768800)
Supplement: Supplementary Table 1 — Baseline characteristics of the SEER subset (2000–2022). [file Table1.docx]

Supplementary Table 1 Baseline characteristics of the SEER subset (2000–2022)

| Characteristics | | N (%) |  |
| --- | --- | --- | --- |
| Number of cases | | 5000（100.0%） | |
| Age |  |  | |
| ＜65 |  | 3071 (61.4%) | |
| ≥65 |  | 1929 (38.6%) | |
| Sex |  |  |  |
| Male |  | 4196 (83.9%) | |
| Female |  | 804 (16.1%) | |
| Race |  |  |  |
| White |  | 4534 (90.7%) | |
| Black |  | 244(4.9%) |  |
| Asian or Pacific Islander |  | 191(3.8%) |  |
| Other |  | 31 (0.6%) |  |
| Location |  |  |  |
| Cervical |  | 52 (1.0%) |  |
| Upper |  | 235(4.7%) |  |
| Middle |  | 800 (16.0%) | |
| Lower |  | 3913 (78.3%) | |
| Stage | |  |  |
| II |  | 1988 (39.8%) | |
| III |  | 2834 (56.7%) | |
| IVA |  | 178(3.5%) |  |
| Outcome |  |  |  |
| alive |  | 756 (15.1%) | |
| dead |  | 4244 (84.9%) | |
